# P393: Assessment of the practice of sterilization of medical devices in the services of surgery of chu the university hospital Yalgado Ouedraogo (Chu-Yo)

**DOI:** 10.1186/2047-2994-2-S1-P393

**Published:** 2013-06-20

**Authors:** J Zoungrana, IP Guissou, F Zongo, H Dhainsala

**Affiliations:** 1 Health Service Hospital , CHU Yalgado Ouedraogo, Ouagadougou, Burkina Faso

## Introduction

The consolidation of sterilization on the same site is the only way for institutions to maintain internal control of the sterilization process for medical devices.

At the CHU-YO, the services that are the direct users of these services are responsible for the activities relating to the sterilization of reusable medical devices, which poses the problem of the variability in the application of sterilization procedures.

## Objectives

Conduct an inventory of the sterilization of medical services in surgical units of CHU Yalgado having a sterilizing section.

## Methods

Descriptive cross-sectional survey conducted in seven CHUYO surgical services with personnel involved in the sterilization of reusable medical devices. Data collection was conducted through individual interviews with staff, observation of practices and assessment of documentation available in the service.

## Results

A pre-disinfection occurred immediately after surgery in 93.03% of cases. Dilutions of the preparation of decontamination solutions were made randomly. During decontamination, the equipment was totally immersed in only 72.1% of cases. There was insufficient contact time between disinfectant and contaminated equipment in the majority of observations. Cleaning of all equipment including containers was performed in only 4.65%. Drying was done in the open air in two (02) in five (05) other services. The cleaning material consisted of unsterile compresses (36.36%), already used fields (27.27%), sterile gauze (21.21%), clean fields (15.15%). Sterilization processes used: Poupinel (one service); Poupinel and formalin (five services); Poupinel, autoclave, and formalin (one service). No registration of autoclaving parameters (temperature, pressure, time). Lack of procedures on the steps of sterilizing reusable equipment.

## Conclusion

The application of sterilization procedures allow health care sectors to optimize the safety of care for the benefit of patients.

## Disclosure of interest

None declared

